# Roselle Anthocyanins: Antioxidant Properties and Stability to Heat and pH

**DOI:** 10.3390/molecules23061357

**Published:** 2018-06-05

**Authors:** Hai-Yao Wu, Kai-Min Yang, Po-Yuan Chiang

**Affiliations:** Department of Food Science and Biotechnology, National Chung Hsing University, 145 Xingda Rd., South Dist., Taichung City 40227, Taiwan; j490560511@gmail.com (H.-Y.W.); a9241128@gmail.com (K.-M.Y.)

**Keywords:** roselle, anthocyanins, heat, pH

## Abstract

Roselle is rich in anthocyanins and is traditionally used to prepare a bright red beverage by decoction. However, heat treatment and different pH environments are often encountered during food processing, and these factors are often detrimental to anthocyanins. Therefore, it is very important to understand the influence of pH and heat treatment on anthocyanins for the application of roselle. This study determined the antioxidant properties of roselle extract, explored changes in the color and anthocyanin content in different pH environments, and evaluated the thermal stability of roselle anthocyanins using kinetic equations. The results showed that the roselle extract is rich in anthocyanins and has good antioxidant capacity (DPPH IC50 = 4.06 mg/mL, ABTS IC50 = 3.7 mg/mL). The anthocyanins themselves exhibited a certain degree of heat resistance and good color stability in an acidic environment. In contrast, they degraded very quickly and exhibited significant changes in color in a low-acid environment. The activation energy (*E*a) ranges of the anthocyanins in the acidic and low-acid environments were quite different at 55.8–95.7 and 31.4–74.9 kJ/mol, respectively. Thus, it can be concluded that roselle anthocyanins are susceptible to heat treatment in a low-acid environment, affecting their quality and appearance; however, they can serve as a good source of functional ingredients and color in an acidic environment.

## 1. Introduction

Roselle (*Hibiscus sabdariffa* L.) is recognized as a tropical shrub which belongs to the family *Malvaceae*. Roselle can be found in tropical and sub-tropical regions such as India, Indonesia, and Malaysia, among others [1]. The main planting area for the shrub in Taiwan is in the eastern region of the island, which is why roselle is one of the most important crops in Taitung, Taiwan. The roselle calyx is brightly colored and rich in nutrients such as anthocyanins, organic acids, pectin, etc. [2]. The calyces of roselle have been widely used in medicines and foods such as syrup, refreshing drinks, wines, jams, and natural food colorants [2,3]. The leaves or calyces are traditionally prepared in beverage as they are rich in anthocyanins, which have antioxidant properties and are useful in diuretic and sedative treatments [4]. Anthocyanidin is one of the natural water-soluble pigments and is among the derivatives of the flavonoid compounds found in phenolic compounds [2]. The chemistry of anthocyanins has been reviewed extensively. So far, studies have reported that the most common anthocyanins, which include pelargonidin, peonidin, cyanidin, malvidin, petunidin, and delphinidin, are widely present in fruits and vegetables such as grapes, raspberries, roselle, and purple cabbage, among others [5]. Besides their vibrant colors, anthocyanins also have anti-oxidant and bioactive properties linked to certain health benefits; for example, they have properties linked to anti-diabetic, anti-inflammatory, and anti-cancer effects [6,7,8]. More and more people recognize that food not only satisfies hunger but also provides a variety of health benefits [9]. Health foods include a variety of foods that not only provide direct health benefits by reducing the risk of chronic disease in relatively short periods of time, but also foods enriched with vitamins and polyphenols, as well as foods that improve the well-being of consumers [10]. Functional beverages are widely consumed worldwide and are a fast-growing segment of the health foods category. Since consumers have shown an increasing awareness of different health issues, these compounds have increasingly become the focus not only of scientific research but also of marketing considerations. Many studies have pointed out that phytochemical-rich foods, such as the catechins in green tea, the anthocyanins in grape juice, and the flavonoids in citrus fruit juices, among others, have good health effects [11,12,13]. In short, the anti-inflammatory, anti-oxidant, and cancer-preventing properties of such phytochemicals are among their many proposed health-promoting properties [6,11,13]. Anthocyanin-rich plant materials are increasingly being labeled as functional materials in recent years as they contain high amounts of secondary plant metabolites and traditionally constitute a high proportion in the human diet. Meanwhile, the anthocyanin contents of beverages not only influence the potential health effects of those beverages but also their sensory qualities [5].

Thermal processing and pH adjustment are among the commonly used unit operations in the food industry. For example, thermal processing is the most widely used preservation method in industrial beverage production [14]. However, thermal treatment can cause organoleptic and nutritional loss, as well as changes in the levels of ascorbic acid, phenolic compounds, and carotenoids, thereby leading to decreased antioxidant capacity and other effects on bioactivities.

In nature, anthocyanins are extremely unstable and susceptible to degradation by external factors such as pH, temperature, light, oxygen, enzymes, metal ions, and other factors. In addition to affecting food products directly, anthocyanin degradation may also result in the production of aldehyde substances with benzene rings that can affect human health [15]. While thermal processing and pH adjustment are among the commonly used unit operations in the food industry. For example, thermal processing is the most widely used preservation method in industrial beverage production [14]. However, thermal treatment can cause organoleptic and nutritional loss, thereby leading to decreased antioxidant capacity and other effects on bioactivities. Color fading and off-flavor formation limits the shelf life of commercial products containing anthocyanins, in addition to restricting the utilization of anthocyanins for certain applications. Therefore, the aim of this study was to determine the antioxidant properties of the roselle extract, in addition to exploring changes in the color and anthocyanin content of the extract in different pH environments. Finally, the thermal stability of roselle anthocyanins was evaluated using kinetic equations.

## 2. Results and Discussion

### 2.1. Total Anthocyanins and Antioxidant Capacity of Roselle Extract

Recognition of the potential health effects of antioxidants more than twenty years ago stimulated what could be called the “Antioxidant Bandwagon” of research seeking to determine which natural materials contain the highest levels of the most active antioxidants, the addition of antioxidants to beverages and other forms of foods, prophylactic and therapeutic medical applications, and hyper marketing [16]. Polyphenols are among the well-known antioxidants. They transfer an electron to free radicals, which thus become stable as their electrons are paired. This prevents damage to cells and tissue caused by oxidant stress. Consequently, a diet that is rich in polyphenols could potentially modulate certain secondary physiological effects of oxidant stress, prevent obesity, or optimize the treatment of diabetes [8]. In the present study, dehydrated roselle calyces were found to have a total polyphenol concentration (TPC) of about 683.13 mg gallic acid equivalent (GAE)/100 g. There is a positive correlation between the total content of phenolic compounds and the antioxidant activity of an extract, and over 95% of the antioxidant capacity of extracts is due to their phenolic components [17,18].

Using the pH-differential method, we were able to determine that the total anthocyanin content (TAC) of dehydrated roselle calyces was 361.99 mg CGE/100 g. In other studies, the TAC of different varieties of black rice has been reported to range between 4.1 and 256.5 mg/100 g [19]. It can thus be seen that roselle is a good source of anthocyanins. In addition, many researchers have pointed out that roselle and its extract possess functional properties that can be used to develop new products with additional nutritious characteristics that may provide health benefits to people [1,2]. At the same time, numerous in vitro antioxidant assays have been developed to measure radical scavenging activity, for example, 1,1-diphenyl-2-picrylhydrazyl (DPPH) radical scavenging activity, 2,2′-azino-bis(3-ethylbenzothiazoline)-6-sulfonic acid (ABTS) radical scavenging activity, and ferric ion reducing antioxidant power (FRAP), such that various kinds of oxygen radical absorbance capacity assay (ORAC) are now household words in scientific and health food publications and on internet websites [16]. The abundant pigments in roselle are responsible for its red color and are the main source of its antioxidant capacity. In this study, the antioxidant capacities of roselle extract were determined by measuring its DPPH radical scavenging activity, ABTS radical scavenging activity, and FRAP.

**Figure 1:**
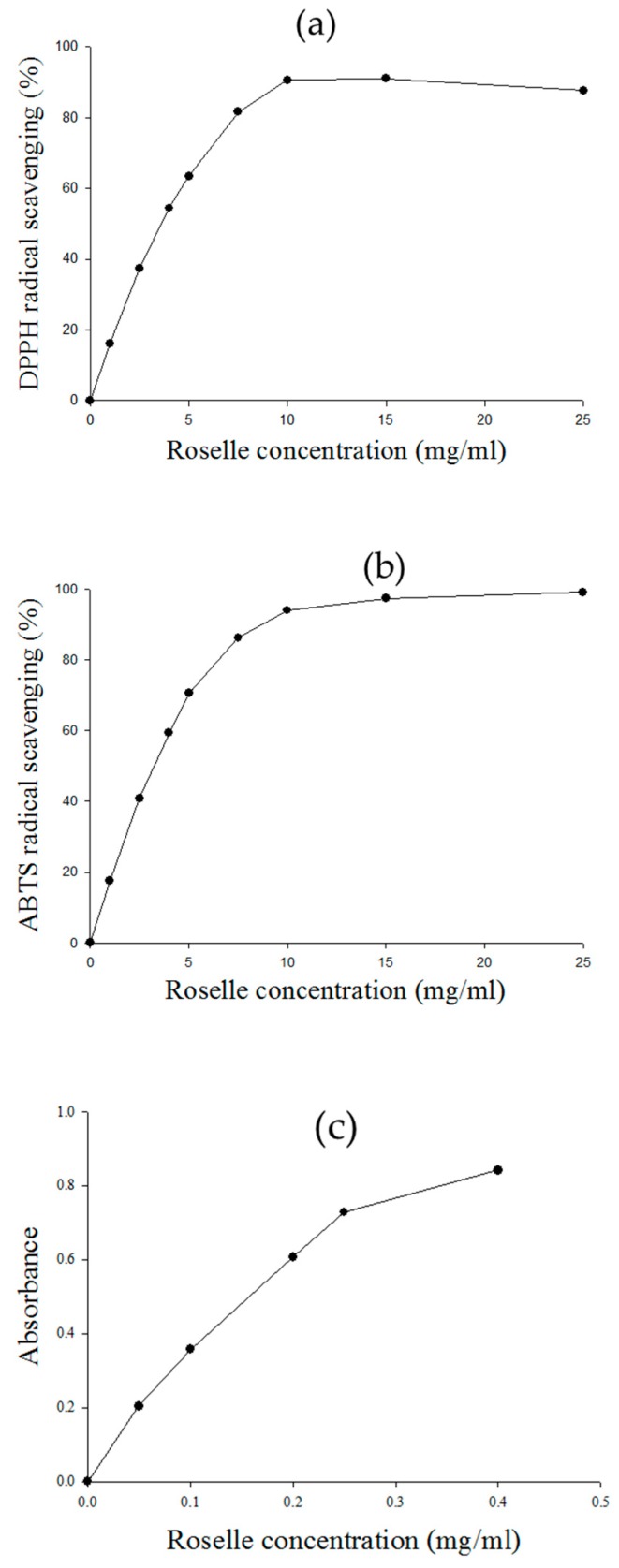
The antioxidant capacity results for the roselle extract of (**a**) DPPH radical scavenging; (**b**) ABTS radical scavenging; (**c**) FRAP.

Figure 1a–c represent the DPPH radical scavenging activity, the ABTS radical scavenging activity and ferric ion reduction antioxidant power, respectively. And their results collectively indicate the antioxidant capacity of the sample. As shown in Figure 1a, the DPPH radical scavenging activity was 20–60% when the sample concentration was 1–5 mg/mL. When the sample concentration reached 7.5 mg/mL, the DPPH radical scavenging activity was above 80%. The calculated IC50 values of the roselle extract and Trolox (6-hydroxy-2,5,7,8-tetramethylchroman-2-carboxylic acid) in the DPPH assays were 4.06 and 0.05 mg/mL, respectively. The IC50 value, defined as the concentration of the extract required for 50% scavenging of radicals under the experimental condition employed, is a parameter widely used to measure free radical scavenging activity. A smaller IC50 value corresponds to a higher antioxidant activity. The IC50 values of the obtained bilberry, blackberry, strawberry, and raspberry pomace extracts were 4.0, 1.7, 3.8, and 4.0 mg/100 mL, respectively [20].

From the ABTS assays, it could be seen that the ABTS radical scavenging activity was 20–80% when the sample concentration was 1–7.5 mg/mL. The calculated IC50 values of the roselle extract and Trolox in the ABTS assays were 3.7 and 0.07 mg/mL, respectively. DPPH is hydrophobic so its reactions must be run in organic solvents. The previous literature has reported that DPPH reactions are mostly attributable to hydrogen atom transfer. In contrast to DPPH, ABTS^+^ is water-soluble, so it can reflect antioxidant capacity in non-organic solvent environments [16]. The ABTS^+^ assay can be used to assess whether antioxidants are hydrogen atom transfer-dominant or single electron transfer-dominant in their reactions, and it can be used to compare changes in the same antioxidant during processing or storage. For example, ABTS^+^ has been used to monitor changes in tocopherol activity after heat exposure in frying oils and in the extrusion of packaging films and to determine the loss of antioxidant activity in strawberries dried with different methods [21,22].

Anthocyanins have been demonstrated to have high antioxidant capacity. In particular, the level of hydroxylation on the 3′ and 4’ positions of the B-ring structure is a fundamental determinant of their radical scavenging activity [18]. In the FRAP assays, the roselle extract exhibited good ferric ion reducing antioxidant power (33.98 mg Trolox equivalents (TE)/100 g sample). Salvador Fernández-Arroyo et al. [23] measured the antioxidant capacity of H. sabdariffa aqueous extract, and the experimental data showed that the values obtained in the FRAP assay for H. sabdariffa aqueous extract doubled those obtained for olive leaf extract. The high FRAP value of the H. sabdariffa aqueous extract could be explained through the reported efficacy of chlorogenic acid and its derivatives, which are the main compounds in H. sabdariffa aqueous extract, as reductants [24].

### 2.2. Color Stability

Anthocyanins are one of the most commonly utilized water-soluble natural colorants because they exhibit vibrant colors that range from red to blue. The color of anthocyanins is strongly dependent on the pH of the surrounding aqueous phase [5]. Relatedly, the fate of anthocyanins during the production of beverages is determined by countless factors, and all of these factors need to be taken into consideration to optimize beverage production processes. Table 1 shows the color changes of roselle extract in the pH range of 1–7 before heating. It can be seen that the color of the extract varied in the different pH buffers, gradually shifting from dark red to light red at pH values of 1–4. When the solution had a pH of 5, the extract presented as nearly colorless, whereas its color changed to blue at a pH of 7. The intensity of the acidity of a food is expressed by its pH value. The pH of a food is one of several important factors that determine the survival and growth of microorganisms during processing, storage, and distribution.

Due to the different composition of food raw materials, the pH value of the beverage will different, so the heat sterilization conditions are not the same. Low-acid foods (pH greater than 4.6 and less than 7.0) need a higher sterilization temperature than acidic foods (pH of 4.6 or below). The purpose of this study was to investigate whether the anthocyanin in roselle is suitable as a nutrient additive and pigment source in beverages. Therefore, we divided the pH range discussed in this study into an acidic environment and a low-acid environment.

**Figure 2:**
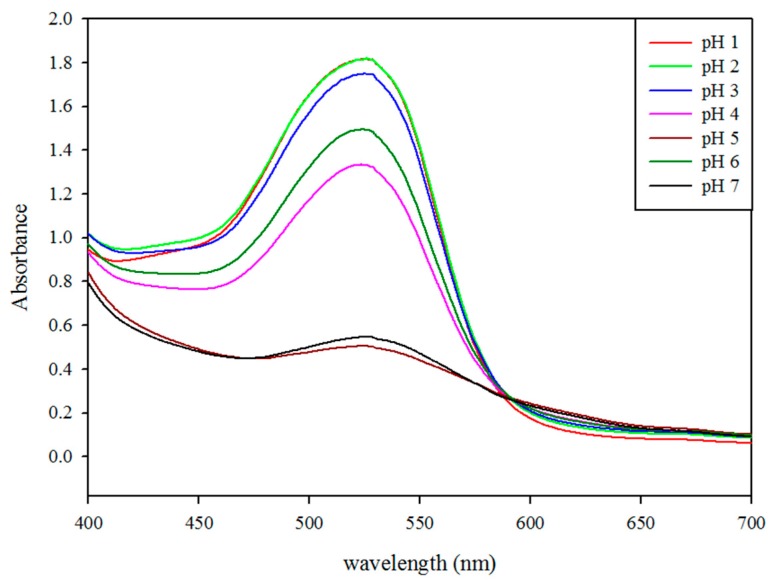
UV-vis spectra of extracts in different buffer solutions ranging from pH 1 to 7.

The UV-visible spectra of roselle extract in different pH buffers were recorded, as shown in Figure 2. When the extract was placed in the acidic environment, the maximum absorption peak was obtained at 520 nm, and the absorbance gradually decreased with increases in the pH value. As the pH increased, the maximum absorption peak showed a slight shift to the right, which was accompanied by a decrease in the maximum absorbance. The bright red color of the roselle extract under certain conditions is due to the main anthocyanins present in the calyces of the plant: delphinidin 3-*O*-sambubioside and cyanidin 3-*O*-sambubioside [25]. In this study, the a value at the initial time was decreased as the pH was increased from 1–6 (Table 1). As the heating time was increased, the a value (redness) of the extract in the acidic environment was decreased, while the b value (yellowness) was increased over the same period of time (data not shown). The main reason for these changes was the occurrence of anthocyanin cleavage and the Maillard reaction [26]. In contrast, the changes in the a value and b value of the extract in the low-acid environment lacked any regularity, possibly because the anthocyanin degraded rapidly and produced numerous degradation products. When the extract was heated in a different pH buffer, the color became unpleasant. Furthermore, as the temperature was increased, the more obvious the above situation was.

The value of the total color difference (ΔE) can express the difference in color of an extract from before heating to after heating in different environments. The larger the value, the more obvious the change in color from before heating to after heating. The results of ΔE of the extract with different heating times and temperatures in different buffer environments are shown in Table 2. It can be seen that the color of the extract changed by varying degrees under heating in different buffer environments. It was pointed out by Kim et al. [27] that human eyes can distinguish a color difference when ΔE > 12. Thus, we can ascertain that when heated at a lower temperature (70 °C), the color of the extract in the acidic environment remains relatively stable. We can draw this conclusion because the color change after 2 h of heating was very small. In contrast, when the extract was placed in an environment with a pH of 7, the color changed obviously even when low temperature heating was applied. The reason for this change is mainly due to the degradation of anthocyanins during heating. When the extract was heated at 70, 80, and 90 °C for 30 minutes at a pH of 7, the residual rates were 29.10, 21.48, and 17.24%, respectively. When the extract was heated at 70, 80, and 90 °C for 2 h at a pH of 2, meanwhile, the residual rates were 87.69, 77.79, and 60.21%, respectively.

The current interest in natural antioxidants from plant sources has become substantial, particularly with respect to bioactive antioxidants such as polyphenols and flavonoids. Roselle anthocyanins have not only good antioxidant properties, but also bright red color, which is suitable as a source of natural pigments in food. However, the low-acid foods need higher temperature and longer time to sterilize, which will cause the degradation of anthocyanins. Meanwhile, the cleavage of an anthocyanin leads to colorless compounds, and polymerization is accompanied by browning [28]. This is what we need to pay attention to.

### 2.3. Thermal Kinetic Degradation

**Figure 3:**
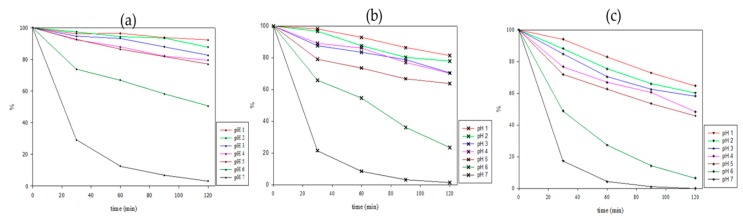
The anthocyanins residual rate (%) during heating at (**a**) 70 °C, (**b**) 80 °C, and (**c**) 90 °C in different pH buffer solutions.

The thermodynamic parameters allowed a deeper understanding of the thermal degradation kinetics of anthocyanins to minimize undesired degradation and to optimize quality of foods. Figure 3 shows the changes in anthocyanin content when the extract was heat-treated in different pH environments. The anthocyanin degradation increased with increasing temperature and time, and the trend of degradation was more obvious with increasing time. After being heated at 70 °C for 2 h in a pH 7 environment, there was almost no anthocyanin content left in the solution, while under heating at 70 °C for 2 h in the acidic environment, the anthocyanin residues still exceeded 80%. Increasing the temperature of the heat treatment is more detrimental to the anthocyanin, but heating at a higher temperature (90 °C) for 2 h in the acidic environment still allows for more than 50% of the anthocyanin content to remain. In this study, the thermal degradation of roselle anthocyanins in buffers with pH values of 1–7 buffer followed first-order reaction kinetics (R^2^ > 0.9). These results were similar to those of previous research indicating that the degradation of anthocyanin follows a first-order model [29].

The first-order reaction rate constants (k), half-life values of anthocyanins (t_1/2_), and *E*a values for a defined temperature range are shown in Table 3. It is clear from Table 3 that as the temperature and pH were increased, the k values increased. The greater the k value, the faster the reaction rate and the faster the degradation of anthocyanins. As expected, the degradation was dependent on temperature and pH, being faster at high temperatures and in low-acid environments. Table 3 shows that the t_1/2_ values for anthocyanin degradation were 1155.2, 385.1, and 182.4 min in a pH of 1 at 70, 80, and 90 °C, respectively, while the t_1/2_ values for anthocyanin degradation were 13.5, 19.4, and 24.8 min in a pH of 7 at 70, 80, and 90 °C, respectively. As the temperature and pH were increased, the t_1/2_ values decreased in a manner consistent with faster reactions accompanied by higher k values. Aurelio et al. [30] previously reported that t_1/2_ values of anthocyanins in a roselle infusion were 11.5, 7.22, and 3.21 h at 70, 80, and 90 °C, respectively. These results are similar to those for the extract in this study at a pH of 2, while the original pH of the extract was about 2.2. 

In the study by Aramwit et al. [31] on the stability of mulberry (*Morus alba*) anthocyanins at 40, 50, and 70 °C, the authors found that the anthocyanin content was significantly decreased after exposure to heating at 70 °C, and they thus advised that mulberry fruit extracts be processed at a temperature lower than 70 °C, a view that supports our own findings. Moreover, Wang and Xu [32] reported that the t_1/2_ values for anthocyanin degradation in blackberry juice were 16.7, 8.8, and 4.7 h at 60, 70, and 80 °C, respectively. Meanwhile, Cemeroğlu et al. [33] reported that the t_1/2_ values for anthocyanin degradation in sour cherry concentrate were 24.0, 10.9, and 4.4 h at 60, 70, and 80 °C, respectively. Comparing these results indicates that anthocyanins from different sources were susceptible to high temperatures. Results from previous research show that when the heating temperature is kept below 80 °C, the rate of anthocyanin degradation is decreased [12].

In general, *E*a is used to describe the energy required to reach the active state of a reaction [34]. In this study, the *E*a range of the roselle extract in the acidic environment was 55.8–95.7 kJ/mol, while its activation energy range in the low-acid environment was 31.4–74.9 kJ/mol. In the acidic conditions, the activation energy decreased with increasing pH, and the activation energy was lowest at a pH of 4 (*E*a = 55.8 kJ / mol), which is similar to the activation energy of grape skin anthocyanins at pH 3.7 (*E*a = 51.0 kJ/mol) in other studies [35]. The high *E*a values revealed that the anthocyanin degradation was slower at the same temperature. These results proved that roselle anthocyanins are more stable in acidic environments than in low-acid environments. Therefore, roselle is suitable for addition to acidic beverages, in which it not only has good functionality but also a pleasant color.

## 3. Materials and Methods

### 3.1. Materials and Reagents

Dehydrated roselle (*H. sabdariffa* L.) calyces were purchased from the Taitung County Farmers’ Association, Taiwan. ABTS, DPPH, Trolox, and gallic acid were purchased from Sigma Chemical Co. (St. Louis, MO, USA). All other analytical grade chemicals and ethanol were purchased from Echo Chemical Co., Ltd. (Miaoli, Taiwan).

### 3.2. Roselle Extract Preparation

Dehydrated roselle calyces were ground into powder with milling, then mixed with 30% (*v*/*v*) ethanol in a 1:20(*w*/*v*) ratio, which was followed by extraction at 75 °C for 20 min in a water bath. After that, the ethanol extract was filtered and stored in a dark bottle at 4 °C until use.

### 3.3. Antioxidant Assays

#### 3.3.1. Total Anthocyanin Concentration (TAC)

Monomeric TAC was determined using the pH differential method [20]. Briefly, two solutions were prepared at pH 1.0 (0.025 M KCl) and pH 4.5 (0.4 M CH_3_COONa). The extract samples were diluted accordingly to be within a measurable absorbance range. The two samples diluted to pH 1.0 and pH 4.5 were then shaken and equilibrated in the dark for 15 minutes. Next, the absorbance values of the samples were measured by spectrophotometer (Hitachi, Tokyo, Japan) at 700 nm (A_700_), and the maximum absorption wavelength (A_λ vis-max_) of each was used to calculate their respective TAC values. Each TAC value was expressed as mg cyanidin-3-glucoside (C3G) equivalents per liter of concentrate according to the following equations:

Absorbance (A) = (A_λ vis-max_ − A_700_) pH 1.0 − (A_λ vis-max_ − A_700_) pH 4.5

TAC (mg/L) = (A × MW × DF × 1000)/(ε × l)

MW: the molecular weight, calculated as cyanidin-3-glucoside (449.2); DF: the dilution factor; l: the cuvette radius, 1 cm; ε: the molar absorptivity, calculated as cyanidin-3-glucoside (26,900).

#### 3.3.2. Total Polyphenol Concentration (TPC)

The total phenolic content values were determined colorimetrically using the Folin–Ciocalteu method [20]. One mL of sample (diluted 1:20 (*v*:*v*) with 30% ethanol) was mixed with 1 mL of Folin–Ciocalteu reagent at room temperature. After waiting for 3 min, 0.1 mL of sodium carbonate (10% *w*/*v*) was added to adjust to the optimum pH for the reaction. The mixture was vortexed and incubated at room temperature for 1 h, and then the absorbance was measured by spectrophotometer at 735 nm. Gallic acid was used as a standard, and the total phenolic content was expressed in mg gallic acid equivalents (GAE) per liter of concentrate. A mixture of water and reagents was used as a blank. All analyses were done in triplicate (*n* = 3).

#### 3.3.3. DPPH Method

The DPPH assay was conducted according to the method of previous research [20] with some modifications. A working solution of DPPH was prepared daily, and the concentration of 0.1 mM of diluted sample was mixed with 1 mL of DPPH. The mixture was then vortexed and left to stand for 10 min in a dark place at room temperature. The absorbance was then measured spectrophotometrically at 517 nm. Trolox was used as a reference standard. The percent of reduction of DPPH was calculated by the formulation:

%DPPH reduction = (1 − As/Ac) × 100

where Ac = absorbance of a control, As = absorbance of sample.

#### 3.3.4. ABTS Method

The ABTS radical scavenging activity was assayed as previously reported [22]. ABTS radical cation (ABTS ^+^) stock solution was prepared by mixing ABTS (7 mmol/L) with potassium persulfate (2.45 mmol/L). The mixture was then allowed to stand in the dark for 12–16 h at room temperature prior to use. The stock solution was subsequently diluted with deionized water until the absorbance was 0.7 ± 0.02 at 734 nm. The diluted sample (0.1 mL) was mixed with 2 mL of ABTS^+^ working solution. The mixture was vortexed and left to stand for 10 min in a dark place at room temperature, and then the absorbance was measured spectrophotometrically at 734 nm. ABTS radical scavenging activity was calculated in the same way as DPPH radical scavenging activity.

#### 3.3.5. FRAP Method

The ferric reducing ability of the extracts was determined using the method of previous research [23]. To perform the assay, 0.2 mL of FRAP reagent and 1 mL of the diluted sample were mixed. The absorbance was then measured at 593 nm by spectrophotometer after it was left to stand for 4 min in a dark place at room temperature. The FRAP reagent working solution was used as the blank. The ferric reducing ability was expressed as mg 100  g^−1^ TE.

### 3.4. Effect of Temperature and pH on the Roselle Extract

#### 3.4.1. Extract in Different pH Buffers

Samples of roselle extract and citrate–phosphate buffer solutions with different pH (1–7) were mixed in a 1:4 ratio, and then put in a water bath (70, 80, or 90 °C) with shaking (90 rpm). The samples were removed from the water bath at 30 min intervals and cooled rapidly to determine the anthocyanin content and color. Three replicates per treatment group were performed.

#### 3.4.2. Wavelength Scanning

Samples of roselle extract and buffers with different pH (1–7) were mixed in an appropriate ratio, then measured with a spectrophotometer (Hitachi U-2800-A, Hitachi, Tokyo, Japan) for wavelength scanning (400–700 nm) after 15 min.

#### 3.4.3. Color Measurement

The samples were placed into a color meter (Color meter, NE-4000, Nippon Denshku Industries Co., Osaka, Japan) with Hunter LAB coordinates (L, a, b) to determine their lightness (L), redness (a), and yellowness (b). The instrument (transmittance, C illuminant, 2° observer) was calibrated with black and white (X = 92.81, Y = 94.83, Z = 111.71) reference tiles and then the color difference (ΔE) of each sample was calculated.

ΔE = [(L − L_0_)^2^ + (a − a_0_)^2^ + (b − b_0_)^2^]^1/2^

where L_0_, a_0_, b_0_ is the value of L, a, b at time zero.

### 3.5. Kinetics of Degradation

The degradation reaction of anthocyanins follows the first-order reaction [29], and the rate of the degradation reaction is related to the anthocyanin content in the heating process. The equation can be expressed as follows:

ln(C_t_/C_0_) = −k × t

t_1/2_ = −ln 0.5 × k^−1^

C_0_: initial anthocyanin content. C_t_: heat t time anthocyanin content; k: reaction constant. t: heat time (min). t_1/2_: the half-life.

Activation energy calculation: The Arrhenius equation can be expressed as the relation between the reaction rate constant and the temperature.

k = A × e^−*E*a/RT^

lnk = lnA − (*E*a/RT)

A: proportional constant of the reaction. *E*a: the activation energy (kJ/mol); R: the gas universal constant (8.314 J/mol/k). T: the temperature (K).

The *E*a value was calculated from the slope of the straight lines using a linear regression procedure of the SigmaPlot (SigmaPlot 10.0 Windows version, SPSS Inc., Chicago, IL, USA).

### 3.6. Statistical Analysis

The experimental data were subjected to a two-way analysis of variance, and the means were compared by Duncan’s multiple range test at a *p* < 0.05 significance level using SAS (SAS Inst., Inc., Cary, NC, USA). The parameters of the kinetic models and the Arrhenius equation were estimated by either the linear regression procedure or non-linear regression iterative procedure of SigmaPlot (SigmaPlot 10.0 Windows version, SPSS Inc.). All analyses were done in triplicate (*n* = 3).

## 4. Conclusions

In this study, we determined the total anthocyanin content, antioxidant capacity, and changes in the color of roselle extract during the roselle extract degradation process. The anthocyanin degradation followed first-order reaction kinetics. A higher degradation rate was observed in low-acid environments at higher temperatures. However, anthocyanin content was not only affected by temperature but also by pH. Roselle anthocyanins have a certain degree of heat resistance in acidic environments and thus have potential for application in acidic beverages. For example, it can be used as a nutritional supplement and pigment in drinks. Studies such as this one are important for providing insights into the application of roselle in industrial production.

## Figures and Tables

**Table 1:** The value of lightness (L), redness (a), and yellowness (b) of the extracts in different pH buffer solutions.

	pH 1	pH 2	pH 3	pH 4	pH 5	pH 6	pH 7
L	54.34 ± 0.03	58.32 ± 0.04	57.72 ± 0.03	69.36 ± 0.04	78.83 ± 0.07	75.25 ± 0.09	46.43 ± 0.12
a	57.85 ± 0.14	57.72 ± 0.06	50.21 ± 0.03	35.88 ± 0.04	16.52 ± 0.05	11.14 ± 0.09	19.26 ± 0.06
b	26.70 ± 0.03	24.80 ± 0.09	20.18 ± 0.08	12.61 ± 0.02	10.56 ± 0.01	12.46 ± 0.10	6.44 ± 0.17

Data presented are in mean ± SD (*n* = 3).

**Table 2:** Effects of temperatures on the total color difference (ΔE) of the extracts in different pH buffer solutions.

Temperature (°C)	Time (min)	pH 1	pH 2	pH 3	pH 4	pH 5	pH 6	pH 7
70	30	1.78	0.88	2.83	4.11	3.46	6.57	30.03
	60	0.65	1.33	3.81	4.56	3.57	7.42	34.79
	90	0.68	1.21	4.25	5.35	4.49	8.54	36.67
	120	0.69	1.97	5.31	5.73	5.01	9.32	36.47
80	30	1.29	1.00	3.14	2.67	4.83	7.48	24.56
	60	1.49	2.02	5.17	3.70	6.14	9.86	27.05
	90	14.46	3.32	6.71	5.53	7.02	11.75	32.84
	120	17.08	4.50	8.92	6.66	7.54	13.20	33.51
90	30	3.51	2.19	12.09	10.07	8.10	10.26	22.76
	60	6.45	4.96	18.02	12.13	9.10	12.45	22.05
	90	10.27	7.37	22.62	14.87	10.17	12.26	22.36
	120	13.93	13.41	26.03	15.41	11.76	16.47	25.96

**Table 3:** Effects of temperatures on the k, t_1/2_, and *E*a values of anthocyanin degradation in different pH buffer solutions.

pH	Temperatures (°C)	k (min^−1^)	t_1/2_ (min)	*E*a (kJ/mol)	Arrhenius Equation (R^2^)
1	70	6.0 × 10^−4^	1155.2	95.7	y = −11509x + 26.18 (0.991)
	80	1.8 × 10^−3^	385.1		
	90	3.8 × 10^−3^	182.4		
2	70	1.0 × 10^−3^	693.1	75.6	y = −9090.3x + 19.62 (0.996)
	80	2.3 × 10^−3^	301.4		
	90	4.3 × 10^−3^	161.2		
3	70	1.4 × 10^−3^	495.1	61.6	y = −7411x + 15.05 (0.998)
	80	2.7 × 10^−3^	256.7		
	90	4.6 × 10^−3^	150.7		
4	70	1.9 × 10^−3^	364.8	55.8	y = −6709.4x + 13.24 (0.969)
	80	2.8 × 10^−3^	247.6		
	90	5.6 × 10^−3^	123.8		
5	70	2.2 × 10^−3^	315.1	53.6	y = −6445.4x + 12.66 (0.998)
	80	3.6 × 10^−3^	192.5		
	90	6.2 × 10^−3^	111.8		
6	70	5.3 × 10^−3^	130.8	74.9	y = −9000.3x + 21.00 (0.999)
	80	1.2 × 10^−2^	59.2		
	90	2.3 × 10^−2^	30.8		
7	70	2.8 × 10^−2^	24.8	31.4	y = −3773.7x + 7.41 (0.984)
	80	3.6 × 10^−2^	19.4		
	90	5.1 × 10^−2^	13.5		
